# Using Data to Keep Vaccines Cold in Kenya: Remote Temperature Monitoring With Data Review Teams for Vaccine Management

**DOI:** 10.9745/GHSP-D-19-00157

**Published:** 2019-12-23

**Authors:** Mercy Lutukai, Elizabeth A. Bunde, Benjamin Hatch, Zoya Mohamed, Shahrzad Yavari, Ernest Some, Amos Chweya, Caroline Kania, Jesse C. Ross, Carmit Keddem, Yasmin Chandani

**Affiliations:** a inSupply Health, Nairobi, Kenya.; b JSI, Boston, MA, USA.; c JSI Research & Training Institute, Inc., Boston, MA, USA.; d Nexleaf Analytics, Los Angeles, CA, USA.; e Ministry of Health, Republic of Kenya, Nairobi, Kenya.; f Nexleaf Analytics, Nairobi, Kenya.

## Abstract

**Background:**

Global vaccination coverage rates have remained around 85% for the past several years. Increasing immunization coverage rates requires an effective cold chain to maintain vaccine potency. Remote temperature monitoring (RTM) technology for vaccine refrigerators has shown promise for improving the ability of supply systems to maintain optimal temperature conditions to ensure potent vaccines reach the end users.

**Methods:**

A pilot study of RTM technology and data use teams was implemented in 36 study sites in Kenya. Data were collected at baseline and endline points over a 3-month baseline and 7-month implementation period. Data included 44 qualitative interviews, process logs, meeting minutes from data use team meetings, and quantitative temperature and power data from the RTM devices.

**Results:**

The ability of cold chain equipment to maintain World Health Organization-recommended temperatures in study sites improved markedly between the baseline and implementation periods, resulting in an improvement in total time spent in the correct range from 83.9% in the baseline period to 90.9% in the intervention period and an improvement in time spent in the too cold range from 6.5% to 1.5%. Friedman tests revealed that differences in time spent in the correct range and time spent in the too cold range during the course of the study were statistically significant (*P*<.001 and *P*=.04, respectively). Qualitative and quantitative data suggest that this improvement was due to a combination of improved responsiveness to temperature excursions at the facility level, resulting from SMS alarms for temperature excursion periods, and improved ability at the management level to recognize and address recurring problems.

**Conclusion:**

The combination of using RTM technology with a structured data review process by a management team is a promising approach for improving cold chain outcomes. Future research examining the added value of each of the technological and behavioral components separately is needed.

## Abstract (precis)

Using technology to make data visible to stakeholders and giving those stakeholders a framework for analyzing that data for decision making improves cold chain management of vaccines in Kenya.

## INTRODUCTION

Globally, in 2016 more than 5.6 million children died before their fifth birthday, mostly from preventable causes.^1^ Immunization has been recognized as one of the most successful public health interventions, but global vaccination rates have remained stagnant at 85% for the past several years.^2^^–^^4^ The World Health Organization (WHO) estimates that improving vaccine coverage rates could prevent an additional 1.5 million deaths per year.^4^ To achieve the high immunization coverage rates needed, effective cold chain management for maintaining vaccine potency is required.^2^ Remote temperature monitoring (RTM) technology allows for real-time vaccine cold chain equipment (CCE) temperature monitoring and also provides an avenue for CCE data visibility and use. This enables better monitoring of CCE performance. However, few studies exist about how to integrate it into public health supply chains in a way that ensures data are used for action and decisions and to ensure investments are cost-effective.

WHO standards define an adverse heat event as occurring when vaccines experience a temperature above 8°C for a period of 10 hours or more. An adverse freezing event occurs when vaccines experience a temperature below −0.5°C for a period of 1 hour or more, reflecting the greater general sensitivity of vaccines to freezing than to heat events.^5^ WHO guidelines recommend storing vaccines between 2°C and 8°C at all levels of the cold chain because exposure to heat or cold outside that range can adversely affect the immunological properties of the vaccines and thus reduce their potency.^6^ Administering compromised vaccines will not provide the intended immune response to protect the vaccinated client and that, in turn, can prevent countries from effectively reaching their coverage targets.^6^ A number of studies have shown that exposure to temperature extremes within vaccine supply chains is relatively common in both developed and developing countries. As much as 37% of vaccines are exposed to temperatures below the recommended range in lower-income countries, making this a critical issue to address.^7^^–^^13^

Despite the importance of ensuring appropriate temperature ranges for the storage of vaccines throughout the cold chain, relatively few studies have examined the use and effectiveness of temperature monitoring practices or existing studies have found monitoring practices to be substandard, commonly resulting in exposure to temperature extremes.^13^ For example, a study of North West Region, Cameroon, found that only 76% of health facilities examined had a functioning thermometer for their vaccine storage unit, and of those, 20% were experiencing abnormal temperatures at the time of data collection.^14^ In addition, lack of information about what is happening in the vaccine cold chain at the intermediate and facility levels, particularly about the state of functionality of vaccine storage equipment and the exposure of vaccines to temperature extremes at the last mile of the distribution network, is common among many cold chains in developing countries.^15^ As discussed below, Kenya’s vaccine supply chain suffers from many of these same problems.

The National Vaccine and Immunization Programme (NVIP) manages Kenya’s vaccine cold chain. At the central level, NVIP stores the vaccines that UNICEF procures for the Expanded Programme on Immunization (EPI). On a quarterly basis, the central level distributes vaccines to 8 regional stores, and subcounty level vaccine stores collect the vaccines from the regional stores. On a monthly basis, more than 6,900 immunizing health facilities collect their vaccines from the subcounty hospitals.

To gauge the status, strengths, and weaknesses of vaccine management in the NIVP, in November 2013, Kenya used the WHO-UNICEF Effective Vaccine Management Tool to conduct an effective vaccine management assessment.^16^ The tool assesses each level of the immunization supply chain and makes recommendations to address areas of weakness. The 2013 effective vaccine mangement assessment found that the NVIP’s efforts to increase immunization coverage and prevent disease were significantly hampered by compromised vaccine potency resulting from a lack of CCE preventive maintenance and timely repair, outdated equipment inventories, a shortage of spare parts, and poor temperature monitoring by health care workers. These factors, combined with vaccine stock availability issues, hindered Kenya’s efforts to increase immunization coverage and prevent vaccine-preventable diseases.

In addition to the problems noted in the assessment, CCE temperatures at facilities and subcounty stores are manually tracked and recorded using the Fridge-tag 2 (FT2), a continuous temperature monitoring logger. The FT2 has a number of documented problems related to users’ lack of knowledge on its use, how to read and interpret FT2 readings, and how to initiate action in response to temperature excursions.^16^ Lack of temperature data visibility at different levels of the health system further compounds the challenges in Kenya since temperature data at the facility level uses a paper-based recordkeeping system.

RTM technology for real-time recording and reporting of refrigerator temperature data is a promising innovation to increase access to this information. However, as with any technology, RTM technology alone is not enough to ensure optimal outcomes in maintaining ideal temperature ranges. For example, a study in Laos found that although remote reporting of temperature data was successful, additional training was required to enable data managers to effectively use the data and translate it into effective decision making, highlighting the importance of addressing health worker behavior in addition to technical solutions.^15^ Similarly, Comes et al. identified real-time temperature monitoring as a promising technology for transforming the performance of cold chains but concluded that there are major gaps in the research into how information gets used by decision makers in the field to support improvements in the functioning of the cold chain.^17^

Using remote temperature monitoring to record and report temperature data is a promising innovation to increase access to this information.

We hypothesize that cold chain managers in Kenya do not currently have sufficient data to monitor the performance of their cold chain equipment, are not effectively using the data they have, and are not empowered to effectively escalate issues to higher-level decision makers, who lack appropriate visibility into cold chain performance. To address these gaps, we designed our study to assess the effectiveness of combining the use of RTM technology and a problem solving approach with a data use team that included members from multiple administrative levels, including both ground-level implementers and higher-level decision makers. The RTM devices were deployed in service delivery sites to facilitate access to real-time temperature and power availability data of vaccine refrigerators. Service delivery sites in similar geographic areas were overseen by a data use team, which used systematic data use and problem solving approaches for addressing temperature excursions and cold chain equipment malfunctioning. The combined technology and behavioral approach provided insight into how to protect vaccine potency through improved cold chain management practices and equipment performance in Kenya.

## METHODS

### Intervention Description

The study intervention included 2 components to address both equipment and behavior issues: an RTM data collection system and a structured team approach to data review.

The study intervention assessed equipment and behavior issues in cold chain management.

First, RTM devices were installed in 59 refrigerators in 36 health facilities and subcounty vaccine stores located in the intervention area. The RTM system consisted of 2 major parts, the hardware and the dashboard. The hardware was a global system for mobile communication (GSM)—that is, connected to a cellular network—with temperature sensor probe(s) that were placed inside a vaccine refrigerator, with the main body of the device positioned nearby, usually mounted on a wall. The system uploaded temperature and grid power availability data to a server using cellular networks. Every 10 minutes, the system collected and sent continuous temperature data to an online dashboard. When temperature excursions occurred, the systems sent SMS text messages to key personnel and emitted audible alarms. Long battery life (up to 3 days) helped ensure continuous operation in the event of a power outage.

The second part of the RTM system, the dashboard, organized and displayed the collected data through various visualizations and analytics to inform decision making for technicians and managers. Standard visualizations showed each refrigerator’s performance as the percentage of time each temperature probe (and refrigerator) measured in each of 3 temperature bands (below, within, and above the WHO recommended temperature range), as well as the number of alarms recorded by the devices each month. Cold chain handlers previously had country-specific standard operating procedures (SOPs) that detailed how to record data from the standard FT2 devices. We provided them with updated SOPs that differed in that they explained how to respond to temperature excursion alarms from the RTM system, maintain cold chain equipment, and escalate unresolved cold chain issues to the county and national levels to be addressed when appropriate. These SOPs were posted near the vaccine refrigerator to help clinic personnel respond to RTM alerts effectively. Facility personnel were also requested to complete process logs to describe actions taken upon encountering alarms.

The second component of the intervention focused on improving behavior of cold chain personnel and improving data use through data use teams. This was a structured approach to team data review modeled after the logistics control tower approach used by many private sector logistics firms. The approach emphasized a discrete team charged with overseeing the performance of the supply chain, selecting indicators to measure performance, using data to track those indicators on a regular basis, and making decisions to address any problems or performance deficiencies identified. These data use teams were comprised of health facility nurses, subcounty and county biomedical engineer technicians, vaccine depot nurses, EPI logisticians, and health records information officers. A set of key performance indicators, derived from the data produced by the RTM devices, were jointly selected with NVIP. These indicators were monitored on a monthly basis and were disaggregated by county, CCE model, and type of facility for analysis. The key indicators selected included number of excursions (high or low temperatures outside of the acceptable range of 2°C to 8°C), percentage uptime (percentage of total time a CCE spent in the range of 2°C to 8°C), and field holdover time (the average amount of time a vaccine fridge in the field maintained safe temperatures after a power outage).

Having a team with members from multiple disciplines enabled the team to collectively gain a more complete picture of the performance of the supply chain, instead of each member focusing only on the indicators most familiar to them while neglecting others. During the monthly data use team meetings, the teams reviewed performance against key indicators outlined in a jointly established performance plan; identified performance problems; performed root-cause analysis of such problems and brainstormed solutions; and developed or updated the team’s action plan to address these problems. Recognition of achievements and good performance also served to motivate members to continue striving for performance outside of team meetings. RTM data and the RTM performance dashboard featured as a key component of the data review process during team meetings, though additional program indicators such as vaccine coverage were also tracked.

Having a team with members from multiple disciplines provided a more complete picture of the supply chain performance.

### Study Sites

The study was implemented in Isiolo, Kajiado, and Nairobi counties. These counties were selected because they were identified as priority counties by the Ministry of Health under their health systems strengthening work stream, were participant counties in the Reach Every District, Reach Every Child strategy, and had already established data use teams for vaccine supply chain management. Further, these counties were representative of the different climatic conditions and geographies of other counties in Kenya. Across the 3 counties, 36 study sites were selected to include 18 subcounty vaccine stores and 18 service delivery points with high volumes of vaccine administration. With limited resources to implement the RTM devices, these sites were specifically chosen due to their high volume of vaccine throughput, both to maximize the potential effect of the intervention and because such high-volume sites would be the first targets in any eventual wider-scale adoption of the intervention. These sites represented a small minority of total sites in each county, including 6 of the 46 immunizing sites in Isiolo County (13%), 10 of the 174 sites in Kajiado County (6%), and 20 of the 444 sites in Nairobi County (5%); in total, the 36 sites represented 0.5% of the approximately 7,020 immunizing sites in Kenya.

### Study Design

The study used a nonrandomized, pre- and posttest intervention design to determine the efficacy of a combined approach of RTM system implementation for continuous temperature monitoring at the facility and store level with structured data review for action processes by health personnel at multiple levels.

Institutional Review Board approval for this study was not sought, because program leadership, including the principal investigator and supervisors of the implementing team, determined that these activities constituted quality improvement rather than human subjects research. This determination was supported by the focus on a standard programmatic process that would be improved by the RTM concept; the involvement of internal program staff rather than outside evaluators; and the primary goal of informing operational and strategic decision making. Nonetheless, approval of all activities was obtained from the Kenya Ministry of Health, and informed consent was obtained and documented from all subjects interviewed during the pre- and postintervention periods.

### Data Collection and Analysis

Baseline and endline interviews (Supplement) were conducted with EPI personnel, facility in-charges, public health nurses, vaccine depot managers, medical engineering technicians, and health records and information officers. These personnel were involved in either CCE use and management or CCE data performance monitoring at the facility, subcounty, and county levels.

Baseline data were gathered from July to September 2017, including qualitative interviews with 13 total health personnel at study sites. During this time, RTM devices were installed, and the devices recorded and transmitted temperature and power data to the RTM dashboard. During the baseline period, the devices were not configured to send SMS alarms, and the data use team members and facility managers were not provided access to the online RTM dashboard. Qualitative interviews were conducted with 13 EPI staff and cold chain personnel at various levels at each study site to gather information on staff’s knowledge of vaccines and current cold chain management practices. While health workers were trained during the baseline period in using the process logs and new SOPs, they continued to follow protocols outlined in the existing SOPs for monitoring refrigerator temperatures using the standard FT2 loggers and paper charts.

The intervention period ran from October 2017 to April 2018. At the start of the intervention period, the RTM system was activated to begin sending audible and SMS alarms to health personnel for temperature excursions and power outages. Key managers at all levels of the system were provided access to data on the online RTM dashboards. Data use teams were also provided with intensive technical support from October to December 2017 to reinforce the routine structured data use team process, including interpreting and reviewing key cold chain indicators via the RTM dashboard and reviewing key supply chain metrics from Kenya’s District Health Information System 2 (DHIS 2) system already being used by data use teams.

At the end of the intervention period, qualitative interviews were conducted with 31 total health personnel at study sites. If possible, the individuals interviewed during baseline were also interviewed at endline. Questions included similar knowledge and practice questions as at baseline to provide a comparative understanding of knowledge and perceptions before and after the intervention period. Additional questions on their experiences with the RTM devices, experiences with the data use teams, and ongoing challenges in their vaccine management work were included to retrospectively capture RTM and process-related information.

The study documented changes in key metrics related to vaccine refrigerators’ functioning and performance of health worker and cold chain technicians/teams in responding to temperature excursions and maintenance needs. Data used in the analysis came from the baseline and endline qualitative interviews, temperature and power data recorded by the RTM system, written process logs at each site describing alarms and corrective actions taken, and minutes from data use team meetings.

Key themes examined by the qualitative interviews included knowledge about the effect of heating and freezing on vaccines; knowledge and perceptions of the causes of heating and freezing events; recognition of damaged vaccines and the current procedures in managing heat/freeze events and affected stock; and perceived barriers and problems respondents currently face in managing and responding to temperature excursions.

This study measured both the average time spent in excessive temperature zones as well as the number of such heat and freeze excursions (signaled via alarms) that occurred in each refrigerator, based on the temperature and time-series data available in the dashboard. Excursions outside the appropriate temperature range can be indicative of a number of conditions and can help pinpoint appropriate corrective action and redirection of resources. For example, excessive heat alarms may be indicative of frequent power disruptions without appropriate back-up sources of power, or excessive cold alarms may be due to an improperly set thermostat. Excessive cold or heat alarms may also indicate older or poorly functioning equipment that require enhanced preventive maintenance to ensure optimal functionality.

Key indicators from the quantitative temperature data included the percentage of time spent in each temperature band and the numbers of hot and cold alarms calculated by the dashboard according to WHO-defined temperature excursions of a 10-hour period spent hotter than 8°C for a heat alarm or 1 hour spent colder than −0.5°C for a freeze alarm. A Friedman test was run to determine if there were differences in uptime performance during the 10-month study. Pairwise comparisons were performed (SPSS Statistics, 2018) with a Bonferoni correction for multiple comparisons. This nonparametric test was considered most appropriate because our data did not meet critical assumptions around normality, lack of outliers, and sphericity required for validity with a repeated measures ANOVA.

For data analysis, some data from 9 refrigerators were removed due to faulty sensors or refrigerators not in use so as not to skew results. For example, at some sites where a refrigerator was malfunctioning, health staff discontinued use of the refrigerator by unplugging it and removing vaccine supplies to a different refrigerator or facility but did not report the fridge use discontinuation to the study team. However, the RTM device was often left on the refrigerator and continued to transmit data. Where this could be documented, the data from these devices were removed from analysis for the period of time that the refrigerator was not in use. Additionally, 1 of the 18 health facilities selected for the study was removed from analysis entirely as the installation team was unable to find cellular network coverage at the site to enable the RTM device to transmit data.

## RESULTS

### Temperature Data

**FIGURE 1.**
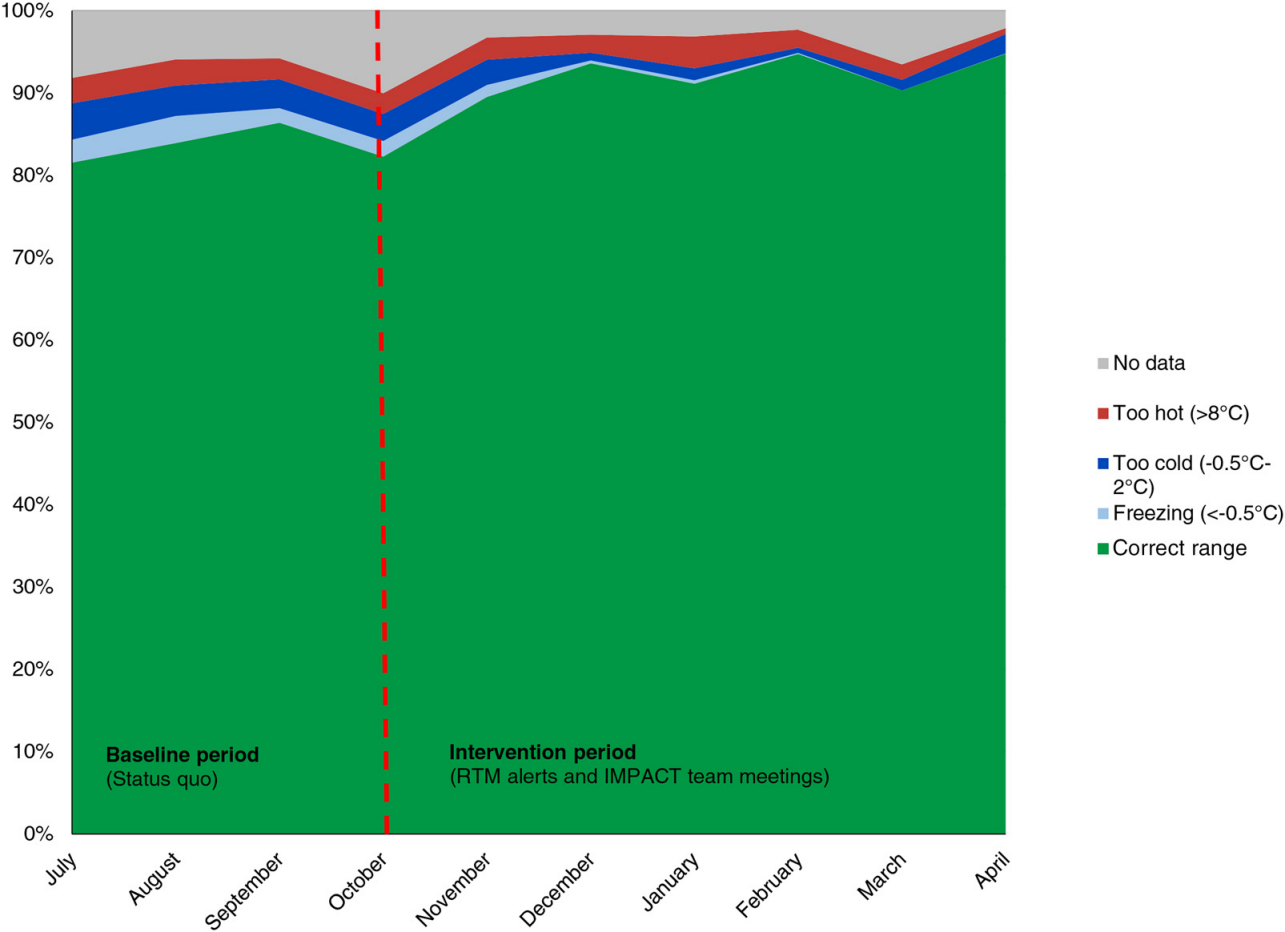
Mean Percentage of Time Spent in Temperature Bands for Vaccine Refrigerators by Month, Baseline (July–September 2017) vs. Implementation (October 2017–April 2018)

Temperature monitoring data revealed a steady improvement in the time that vaccine refrigerators spent in the correct temperature range throughout the implementation period (“uptime”). During the baseline phase, all refrigerators were within the correct temperature range for an average of 83.9% of total time, compared with 90.9% of total time during the intervention phase, as seen in Figure 1. According to the Friedman test, uptime performance was statistically significantly different during the different months of the study, χ^2^(9) = 168.412, *P*<.001. Post hoc analysis revealed that later months of the study were generally not statistically different from each other and earlier months were not statistically different from each other and but earlier and later months were statistically significantly different from each other.

Although there was improvement in the time spent in the ange of being too hot, it was less dramatic, decreasing from 2.9% of total time during baseline to 2.3% during the intervention phase, and a Friedman test revealed this change to not be statistically significant. The most notable result was a sharp decrease in the time refrigerators spent in the combined ranges of being too cold and freezing, from 6.5% during the 3-month baseline phase to 1.5% during the final 3 months of implementation. A Friedman test for these combined ranges revealed that the time vaccines were exposed to cold temperatures was statistically significantly different during the different months of the study, χ^2^(9)=17.663, *P*=.04. This represents a huge reduction in vaccine exposure to inappropriately cold and possibly freezing temperatures.

There was a huge reduction in vaccine exposure to inappropriately cold and possibly freezing temperatures.

**FIGURE 2.**
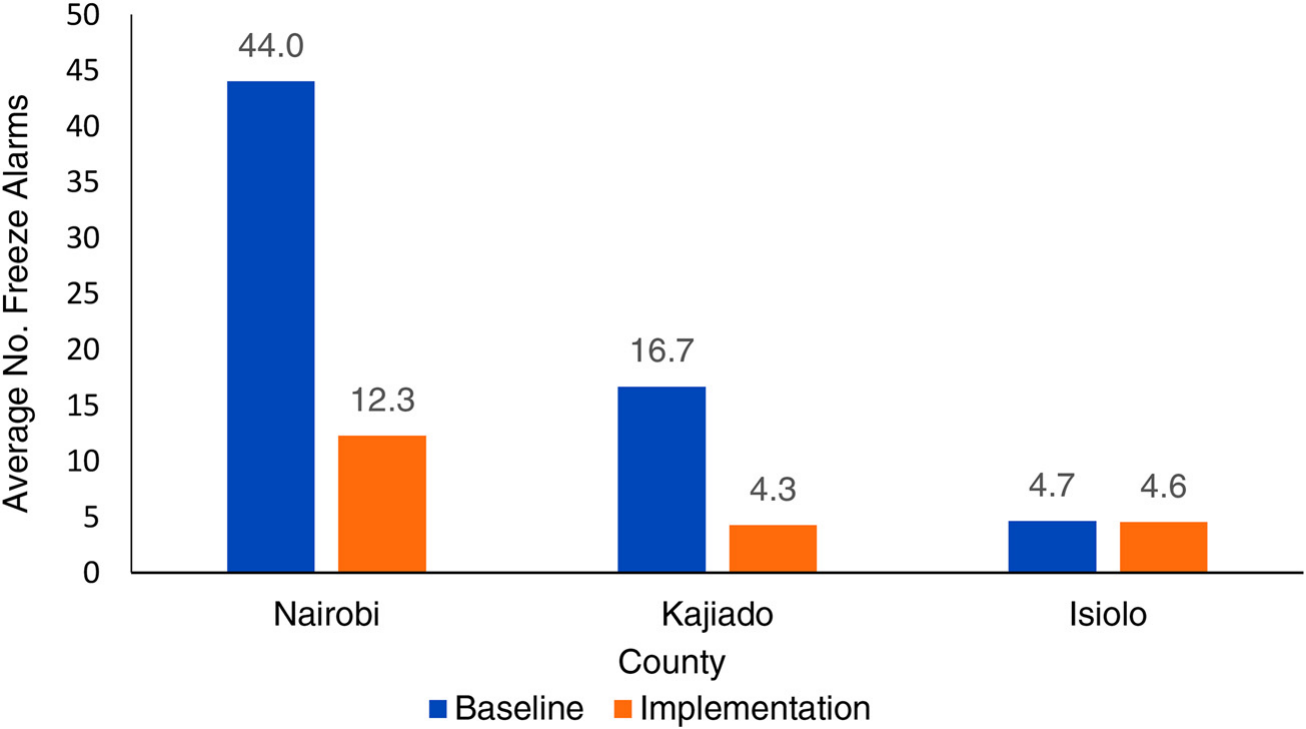
Average Number of Freeze Alarms per Month, by County, Baseline (July–September 2017) vs. Implementation (October 2017–April 2018)

Across all counties between the baseline and implementation period, the time spent in excessive temperature zones decreased. There was a very slight decrease in number of heat alarms, from an average of 16.3 alarms per month during baseline to an average of 15.3 alarms per month during the implementation period. However, across all counties between the baseline and implementation period there was a marked decrease in freeze alarms, from an average of 65.3 alarms per month during baseline to an average of 21.1 alarms per month during the implementation period. Figure 2 summarizes the average number of monthly freeze alarms by county. However, Friedman tests for the differences in alarms showed that these differences do not meet the threshold of statistical significance (*P* =.15 for cold alarms, *P*=.10 for hot alarms).

**FIGURE 3.**
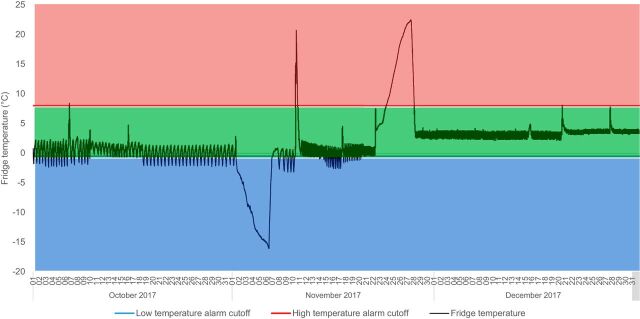
Temperature Oscillation of a Refrigerator with a Malfunctioning Thermostat, Dagoretti Subcounty Store, October 2017 to December 2017

One phenomenon that contributed to the reduction in temperature alarms was the identification and repair of thermostats in fridges identified as problematic during data use team meetings. This can be illustrated in Dagoretti subcounty store. Figure 3 shows the temperature oscillation patterns inside a refrigerator from October through December 2017. In October, the temperature oscillated between 2°C and below freezing, causing 56 freeze alarms, with 90% of total time during that month spent below the 2°C threshold. Data use teams and maintenance logs showed that the problem was identified and fixed during the month of November, after which the same oscillating pattern was observed but the oscillations all happened within the appropriate temperature range. This led to the refrigerator spending 97% of total time in December within the correct temperature zone (with 3% of time at unknown temperature), with no freezing events.

**Figure:**
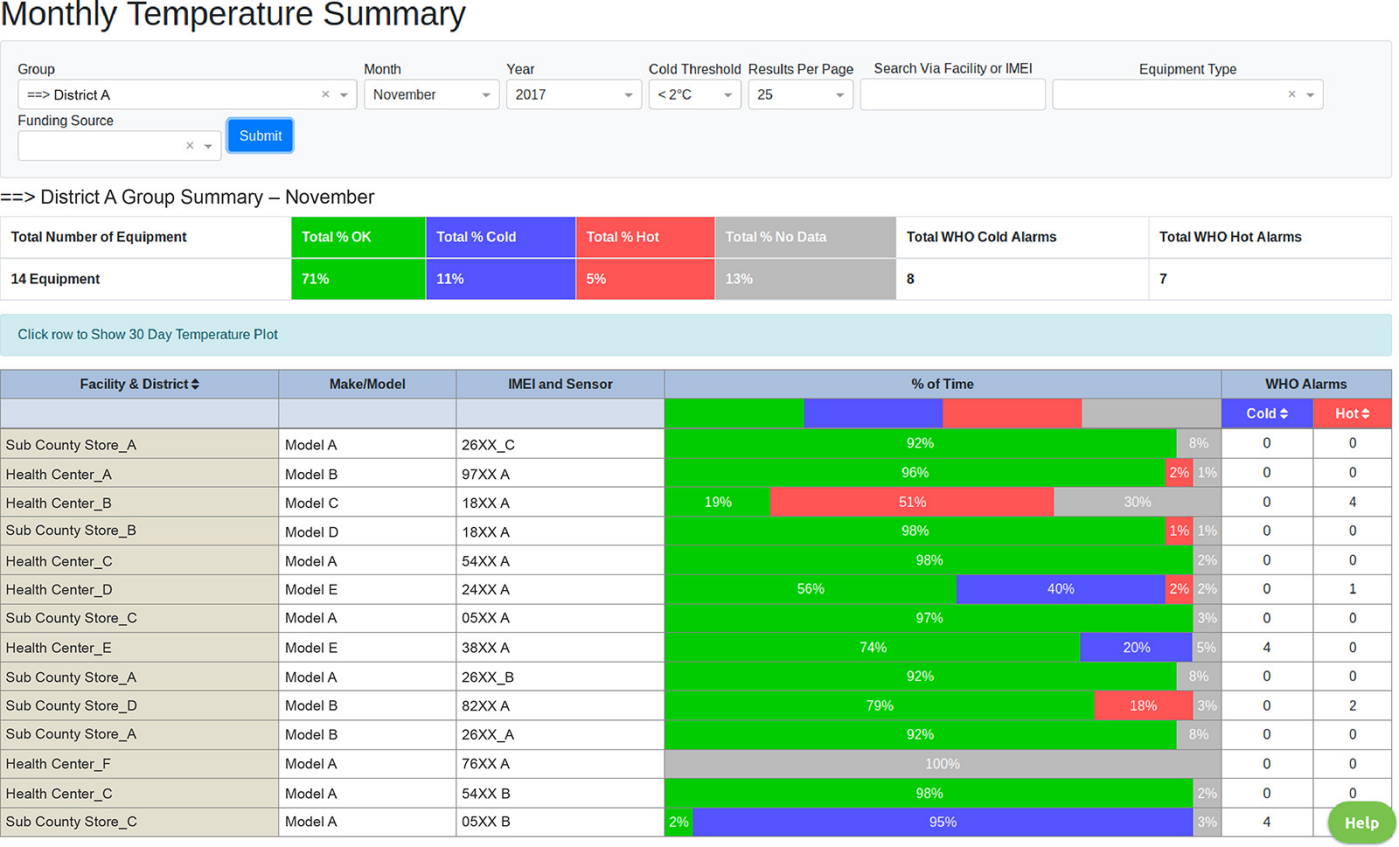
An anonymized screenshot of the remote temperature monitoring dashboard showing equipment performance statistics. © 2019 NexLeaf Analytics

### Behaviors and Practices

Most interviewees at endline noted personnel errors and suboptimal management practices as contributing factors to temperature excursions. Incorrect vaccine storage or packing procedures, such as placing freeze-sensitive antigens in a freezer or in the wrong compartment of a fridge, were commonplace. Other cited vaccine management errors included forgetting to defrost the refrigerator and frequent and unnecessary opening of the refrigerator. Such behavioral issues were also noted at baseline when respondents indicated that providers were often “careless” in excessively opening the refrigerator, thus increasing stress on the equipment and potentially risking heat exposure. These practices were often the result of personnel using the refrigerators for personal reasons, such as to cool a soda on a hot day. Additionally, respondents noted that problems in facilities with poorly performing equipment were exacerbated by these poor management practices.

Beyond these behavior patterns, respondents generally agreed that having the RTM system and receiving alarms helped them to be more aware of and responsive to temperature excursions. In terms of facilitating workflow, most survey respondents agreed that having the RTM devices made their jobs much easier, and almost all respondents described the “alarm-to-action” provided by the RTM devices as having been beneficial to their ability to monitor the state of the vaccine refrigerators and identify and respond to temperature excursions in a timely manner. For example, one facility in-charge noted:

*I think it has changed my work in a way that it’s very easy because even if I’m not in the facility, the moment I get the alarm, I just communicate to one of the staff who is on duty to go and check if the temperatures are going up and if the power is off. It is very easy to manage even if you’re far away and you don’t have to be within the facility.*

Interviewees agreed that having the monitoring system and alarms helped raise awareness and increase responsiveness to temperature excursions.

### Data Use

The RTM system alarms played an important role in enhancing the use of RTM data. However, data use teams played an equally important role in improving cold chain equipment outcomes because the team structure helped to address system or management issues. Interviewees noted that the processes and data used were very helpful, particularly in identifying recurring challenges (e.g., problematic thermostats) and common malpractices regarding cold chain maintenance (e.g., infrequent refrigerator defrosting that affected cold chain performance). As an example, poorly functioning equipment at 1 site had long been a source of concern for Ministry of Health supervisors. Review of RTM data at a data use team meeting revealed that these concerns were justified because this site was a standout poor performer. Having the relevant decision makers discuss this issue together resulted in the refrigerator finally being replaced.

Several respondents also noted that the data use team meetings provided a continual educational forum where common issues and awareness of preventive maintenance practices could be raised and shared. It created a space for all personnel to discuss the data, get insight into the problem by asking questions, and try to find solutions to consistent cold chain failures. As a county logistician stated:

*My opinion is that it’s where we meet and really share the data. When you don’t share the data, it’s like we are in the darkness. The meeting really helps us to see the data, and see whether we’re performing or not and especially on the antigens because in that dashboard for vaccines, we are able to see that the vaccines I am having are they enough, I’m out of stock or overstocked. So all those things we share in the [data use team] meetings and if all the subcounties are together, it helps and we are able to fix a problem.*

Finally, many respondents also embraced the RTM dashboard as a useful technology that helped improve CCE data integrity and accuracy. Through the dashboard, CCE medical engineer technicians and biomedical technicians were easily able to continuously monitor their CCE performance, identify mistakes in their records, and make appropriate corrections. Facility-level nurses also valued the dashboard as revealing the extent of vulnerability in the cold chain. As a nurse stated, “It is an eye opener how vaccines have been exposed to cold and heat excursions.”

## DISCUSSION

Although well-performing equipment is important for cold chain management, other aspects also play a key role in ensuring vaccine potency, including trained personnel, effective and efficient SOPs, and management practices that link trends and priorities in cold chain management with supply chain management and service provision.

The study results indicate that the combined intervention components of the RTM system with the structured data use team approach to data use and problem solving had a direct, positive impact on cold chain management outcomes by ensuring timely action as triggered by SMS alarms and addressing recurring challenges to improve systems overall. Within these results, we outline 3 main conclusions.

### Technology Benefits Can Be Enhanced When Matched With Effective Problem Solving and Decision Making Processes

Qualitative interviews clearly indicated that the SMS alarms provided by the RTM system to health staff served as a cue to timely action and fostered greater awareness of the performance of cold chain equipment. In triangulating with the quantitative data, the improvements in performance appeared to be due to the adoption of improved management practices resulting from data use teams’ enhanced ability to identify recurring prob-lems and take action to address them.

We see this most clearly in the dramatic change in the number of freeze alarms and time spent in the cold temperature range for a few sites. Our results showed that not all refrigerators that spent a significant amount of time below −0.5°C also reported multiple freeze alarms. RTM devices were configured to be more sensitive to cold excursions, as exposure to freezing temperatures has a more immediate negative impact on potency than excessive heat. However, even among freezing alarms, each alarm can indicate a different message about the status of the cold chain. For example, a prolonged freezing period would produce only 1 alarm indicating the equipment stays in the freezing range, whereas multiple relatively short periods of freezing would produce multiple alarms. Although each of these problems may be caused by an incorrectly set or malfunctioning thermostat within the refrigerator, the multiple alarms may indicate a small adjustment is needed rather than a complete thermostat replacement.

The differences in freeze alarms was not obvious to facility-level staff who simply receive the alarms from the system, but data use teams were able to detect this pattern by looking at the time-series data and alarm records used during their data review. Often multiple freeze alarms from a given sensor in a particular month were due to the refrigerator’s thermostat being set too low. This resulted in the refrigerator’s compressor automatically turning on to cool the unit, which would push the refrigerator’s temperature below that −0.5°C mark, thus triggering an alarm after an hour. When the compressor turned off again, the refrigerator would warm up again to just above the −0.5°C mark, resetting the alarm and turning the compressor on again, thus reinitiating the cycle. Consequently, the refrigerators trigger multiple alarms as the temperature cycles back and forth across the −0.5°C mark.

Trends over time, such as these oscillating temperature patterns, were more easily observed through the time-series data available. Combined with the problem solving process during data use team meetings, these problematic thermostats were identified and actions planned and undertaken to adjust or replace them. The reduction in number of freeze alarms is therefore indicative of the ability of the dashboard data and data use teams to identify problematic refrigerators and prioritize them for repair or adjustment.

The composition of the data use teams, which included not just implementing facility-level staff but also engineers and logisticians from the subcounty and county levels with more power to set maintenance agendas, allowed these recurring issues to be escalated and prioritized beyond the facility level where the issues were occurring. Though limitations in spare part availability and travel budgets at times hindered the ability of the health system to address some of these issues, once issues were flagged, they could often be addressed more efficiently, leading to quicker improvements in performance after implementation of the intervention. This was particularly effective in the case of refrigerators that were experiencing regular freezing events due to an incorrectly set thermostat. Once flagged and addressed, a sharp decrease in the time vaccines were exposed to the combined temperature ranges of being too cold and freezing was observed, as outlined above.

### Ability to Triangulate Many Data Sources Is More Likely to Facilitate Holistic Problem Solving

Systems are dynamic and complex with many interrelated issues. Thus, instilling a data use culture for effective cold chain management is facilitated when data are used to address a variety of related system bottlenecks rather than focusing too narrowly on a single issue. Our qualitative results showed that the strategy of expanding existing data use teams that were already looking at coverage and supply chain metrics and adding RTM related metrics enabled teams to triangulate their data, indicators, and results to problem solve more effectively and inform the range of decisions and actions to be taken.

The data use team member feedback during the implementation process also showed that having data available from a variety of sources for multiple indicators encouraged the teams to operationalize the data use concepts. This more robust understanding of the overall situation served as a way to unify the perspectives of the different members of the data use teams since they could each explain their own data in a way that related to overall program performance. Generally, the data use team meetings were described as “very helpful,” a forum to “create teamwork,” and an enabling platform to “discuss issues and evaluate performance.” Some respondents also noted the data use team meetings “helped improve reporting indicators such as vaccine coverage,” “discuss commodity shortages and wastage rates,” and “monitor temperature excursions” to improve conditions.

For example, initially the service providers (nurses) on the team were most interested in service provision statistics provided by existing supply chain data dashboards, since they considered this their primary performance metric. Meetings would begin with reviewing that data first and interpreting data from the RTM dashboard within the context of how it would affect the service provision statistics. They noted that the RTM system performance indicators would have been less interesting and meaningful without that context. In contrast, the technicians on the team were more interested in RTM dashboard data. They are unaffected by service provision data but the RTM data directly affected their decisions and actions. Having both types of data and indicators available, along with members with both perspectives, helped to emphasize the system linkages between the indicators and the drivers of performance challenges. As a result, the team was able to more effectively understand and resolve issues.

Having multiple categories of data also helps the data use teams develop a deeper understanding of the performance of the supply chain. When only a single data source is available and performance is meeting targets, little triangulation of data occurs. Consequently, the data use team is unlikely to take any further action and the value of the forum becomes less evident. Reviewing the different types of data required problem solving and collaboration across team members with different perspectives who are used to working in individual silos, thus promoting shared understanding and shared accountability. The RTM and logistics dashboards capture indicators across these different components and enabled triangulation of data, holistic problem solving, and action planning around cross-cutting programmatic issues.

### Staff-Level Knowledge and Practices Are Key for Long-Term Systemic Change

The qualitative data demonstrated that there are still basic skill and knowledge gaps among both cold chain personnel and EPI staff that were present at both the baseline and endline points. The data use team intervention did not include a training component to address skill and knowledge gaps specifically, which likely affected the effectiveness of the intervention since some health workers may not have been equipped to take corrective actions agreed upon in meetings. For example, at both baseline and endline there was still confusion among several of the respondents about which vaccines were heat or cold sensitive, or the time before the vaccine is considered damaged once the excursion had happened. These knowledge gaps can have important implications for potency. Even if RTM data identified an excursion, without health worker capacity to perform a vaccine vial monitor staging or shake test to determine if the temperature excursion has damaged the vaccine, or even knowledge on basic procedures for managing vaccines within the fridge to minimize damage, an investment in RTM devices will be unlikely to achieve the benefits the technology offers. Many respondents also expressed worry that many cold chain personnel had not practiced these skills or had not had refresher training since their original vaccine management training.

The study results revealed the need for knowledge and skills development (new or refresher) to be included as follow-up actions from data use team meetings and incorporated into the data use team meetings themselves or for a larger initiative to improve knowledge and skills to be implemented as a complement to RTM system implementation and use.

### Limitations

Several limitations affected the results of the study. Limited budgetary allocations to support the continuation of the data use team meetings throughout the implementation period affected the regularity of the meetings and the ability of health personnel to maximize the use of data in their decision making. Althought the data use team meetings were to transition to health facility in-charges meetings starting in January 2018 and supported by local budgetary allocations, not all counties were able to sustain the transport and meeting costs (standard per diem for participants and conference room rental). In some of the Nairobi subcounties, the costs were mitigated by partner resources available to support the meetings, but this support was not consistent across all subcounties, limiting the number of monthly meetings held throughout the intervention period. This affected the ability of the intervention to ascertain the full impact of the behavioral elements supported by the data use team.

The political election period, which started in July 2017 and continued through October 2017, was generally disruptive to the health system and specifically to the study implementation. Several subcounties were unable to hold data use team meetings in October 2017 and health workers were not present at facilities for prolonged periods to monitor or repair cold chain equipment in the event of breakdowns or to address repair needs. Unfortunately, this disruption was coincident with our baseline data collection and may reasonably be expected to have impacted responsiveness to temperature excursion alarms. We were not able to control for this effect in our analysis. The elections also resulted in staffing changes across all 3 counties and at all levels including county directors for health, facility, and EPI staff. This affected the composition of the data use teams. New data use team members were not trained in the approach and were inexperienced in data use team processes, which affected the momentum and effectiveness of some data use teams.

Finally, although teams were trained in the importance of collecting process documentation including the alarm-to-action logs and RTM inventory tools, during the inception training teams were not consistent in completing these. In particular, process logs and meeting minutes were not maintained regularly within any county. Thus, the lack of complete records limited the study team’s ability to cross-check observed patterns of refrigerator performance with documented actions at the facility level. Further, in the absence of action log data, the RTM dashboard cannot discern whether an alarm condition ends due to human intervention or other external factors. This limited the ability of the study to measure the effect of county personnel structures and escalation processes on the time taken to resolve cold chain equipment issues and respond to temperature excursions.

## CONCLUSION

This study reinforces and expands on previous research by Anderson and Comes, which suggested that a combination of RTM data with improved processes for data review and issue management can lead to important improvements in cold chain performance in resource- constrained settings.^15^^,^^17^ The results demonstrated that the real-time alarms for temperature excursions increased staff’s awareness of cold chain performance and their responsiveness to temperature excursions. At the same time, the positive trends in equipment uptime indicate that data use teams played a key role in identifying and prioritizing recurring issues and facilitating longer-term solutions. The study suggests that the combination of various stakeholders in the data use teams and the problem solving structures and processes the teams followed enabled issues that may otherwise have gone unnoticed or remained unresolved to be addressed or escalated more effectively. The observed decrease in regularity of data use team meetings after the suspension of funding for those meetings highlights the importance of ensuring continued support for these teams. Although we believe that the observed results strongly indicate the value of this intervention in terms of potential vaccine losses averted, further study in this area is needed.

As the study was not designed to separate the effect of the RTM system from a structured approach for data review and issue management inherent in the data use team processes, further studies are needed to separate out issues that can be effectively solved by the technology alone versus those that require human or behavioral intervention. However, the study does provide evidence to support combining the use of an RTM system with a structured data use and problem solving team approach as a highly beneficial strategy to improve vaccine cold chain performance throughout the supply chain.

## Supplementary Material

GHSP-D-19-00157-Hatch-Supplement.docx

## Notes

**Peer Reviewed**

**First Published Online:** December 16, 2019

**Funding:** This work was made possible with support from the ELMA Vaccines and Immunization Foundation.
