# Supplementary material for: Using Data to Keep Vaccines Cold in Kenya: Remote Temperature Monitoring With Data Review Teams for Vaccine Management
Source: Glob Health Sci Pract. 2019 Dec 23;7(4):585–97. doi: 10.9745/GHSP-D-19-00157 (PMC6927831; doi:10.9745/GHSP-D-19-00157)
Supplement: GHSP-D-19-00157-Hatch-Supplement.docx [file GHSP-D-19-00157-Hatch-Supplement.docx]

**19-00157 Supplement**

**Facility Staff Qualitative Interview Guide**

| Name of Interviewee |  |
| --- | --- |
| Title |  |
| Category | Facility-in-Charge  SCPHN  SCMET |
| Work Location (County) | Nairobi  Isiolo  Kajiado |
| Work Location (Sub-County) |  |
| Gender | Male  Female |
| Length of time working in current role (complete years) |  |
| Name of Interviewer |  |
| Date of Interview |  |

Questions

1. Does freezing harm vaccines? Which vaccines? Does heating harm vaccines? Which vaccines?
2. How can you tell if a vaccine has been frozen? Exposed to heat?
3. What do you do if you know a vaccine has been frozen? Exposed to heat?
4. At what points during the cold chain do you think a vaccine can be exposure to freezing or heat events? Do you think heat or freeze exposure occurs here (at the facility)?
5. What causes heat or freeze exposure to occur at this point (at the facility) in the cold chain?
6. What equipment changes or training could be done to reduce freezing/heating events?
7. What else could be done to reduce cold-chain freezing/exposure to heat?
8. Do you have current guidelines explaining how to reduce freezing/heating events and what to do if vaccines are frozen or exposed to heat? Describe how you have used these guidelines previously. (If yes, view the guidelines in whatever form they have; take examples or photos of examples if possible and review them)
9. Do you have any current guidelines for cold chain maintenance, repair, or procurement? Describe how you have used these guidelines previously. (If yes, view the guidelines in whatever form they have; take examples or photos of examples if possible and review them)
10. What is the current procedure you use to get assistance if you are unable to resolve a cold chain equipment problem? Can you provide an example from your experience?
11. Did you receive training on these topics?
12. What current problems or barriers do you face in noting and responding to temperature excursions? Cold chain maintenance and repair?
